# Maternal Thyroid Function during the Second Half of Pregnancy and Child Neurodevelopment at 6, 12, 24, and 60 Months of Age

**DOI:** 10.4061/2011/426427

**Published:** 2011-10-24

**Authors:** Jonathan Chevrier, Kim G. Harley, Katherine Kogut, Nina Holland, Caroline Johnson, Brenda Eskenazi

**Affiliations:** Center for Environmental Research and Children's Health (CERCH), School of Public Health, University of California, Berkeley, CA 94704-7392, USA

## Abstract

Although evidence suggests that maternal hypothyroidism and mild hypothyroxinemia during the first half of pregnancy alters fetal neurodevelopment among euthyroid offspring, little data are available from later in gestation. In this study, we measured free T4 using direct equilibrium dialysis, as well as total T4 and TSH in 287 pregnant women at 27 weeks' gestation. We also assessed cognition, memory, language, motor functioning, and behavior in their children at 6, 12, 24, and 60 months of age. Increasing maternal TSH was related to better performance on tests of cognition and language at 12 months but not at later ages. At 60 months, there was inconsistent evidence that higher TSH was related to improved attention. We found no convincing evidence that maternal TH during the second half of pregnancy was related to impaired child neurodevelopment.

## 1. Introduction

The profound deleterious neurodevelopmental effect of maternal and fetal hypothyroidism caused by iodine deficiency has been recognized for more than a century [1]. More recent evidence from experimental and observational studies suggests that even among euthyroid offspring, maternal hypothyroidism and hypothyroxinemia (low thyroxine (T4) with normal thyroid-stimulating hormone (TSH) levels) during early pregnancy may be associated with impaired brain development. Man and colleagues, for instance, reported associations between maternal hypothyroxinemia in early pregnancy and lower scores on neurodevelopmental scales at 8 months, 4 years, and 7 years of age [2–4]. A more recent study by Haddow et al. found reduced scores on tests of intelligence, attention, and visual-motor performance at 8 years of age among children of 48 mothers with untreated clinical hypothyroidism (defined as TSH levels >99.7th percentile or TSH between the 98th and the 99.6th percentile and total T4 < 7.75 *μ*g/dL) at the 17th week of gestation relative to 124 controls [5]. A Chinese study (*n* = 1,268) also found that children of 19 women with hypothyroxinemia (defined as total T4 below the reference range but normal TSH and free T4) and 18 women with subclinical hypothyroidism (high TSH and normal free and total T4) in the first half of pregnancy scored 7.6–10.0 point lower than controls on the mental (MDI) and psychomotor (PDI) development indices of the Bayley Scales of Infant Development [6]. 

Studies have reported reduced scores on cognitive, motor, and language scales even among children of mothers with mild hypothyroxinemia. For instance, in a large population-based cohort study conducted in The Netherlands (*n* = 3,659), Henrichs et al. found 80% increased odds of expressive language delays at 18 and 30 months of age among children whose mother had free T4 levels <10th percentile at 13 weeks' gestation [7]. Pop and colleagues also reported lower scores on the orientation cluster of the Neonatal Behavioral Assessment Scale three weeks after birth (*n* = 204) [8] and a 7.4 point decrease on the PDI at 10 months of age (*n* = 220) [9] in children of mothers with lower free T4 at 12 weeks' gestation. They also found 8–10 point reductions on the MDI and the PDI at 12 and 24 months of age in children of 57 mothers with low-normal free T4 relative to 50 controls [10]. The one study to contradict the above findings did not measure free T4 [11]. Thus, the bulk of the literature points to an association between adverse neurodevelopmental outcomes in offspring and maternal hypothyroidism, hypothroxinemia, and low-normal free T4 levels during the first half of pregnancy.

Evidence suggests that TH of maternal origin may also play a role in fetal development later in pregnancy. This hypothesis was supported by early studies which demonstrated that transfer of radiolabeled T4 and T3 through the placenta continues to occur after the onset of fetal thyroid function [12]. Maternal T4 appears to reach the fetus in significant amounts up until birth, as evidenced by a study conducted by Vulsma et al. [13]. In that study, T4 was detected in the cord blood of 25 neonates with a complete iodide organification defect, a genetic condition that prevents the iodination of tyrosine and therefore inhibits T4 synthesis. T4 measured in cord blood reached concentrations equivalent to 30–60% of the mean values found in full-term fetuses without this condition [14]. Given that a substantial proportion of thyroid hormone reaching the fetus is of maternal origin in the latter part of gestation, it is conceivable that maternal thyroid hormone may continue to affect fetal neurodevelopment during this period. To date, only the studies by Pop and colleagues examined this question in humans and found no relationship between low-normal maternal free T4 (<10th percentile) measured at 24 and 32 weeks' gestation and child neurodevelopment [8–10]. To our knowledge, these results have not been replicated by other groups.

Most studies investigating the association of maternal thyroid function during pregnancy and child cognitive development have focused on hypothyroidism/hypothyroxinemia, perhaps because this condition is more common than hyperthyroidism. Maternal hyperthyroidism is nevertheless a significant condition that affects 0.05–0.2% of pregnancies in the form of Graves' disease; an additional 2-3% of pregnant women are also believed to experience gestational transient thyrotoxicosis [15]. In rats, fetal/neonatal hyperthyroidism causes decreased brain and cerebellar weight as well as abnormal brain development, including an acceleration of neuronal differentiation, a delay in glial cell differentiation and early termination of cell proliferation, resulting in a smaller number of granular and basket cells [16]. In humans, maternal hyperthyroidism during pregnancy has been linked to preeclampsia, fetal loss, premature births, growth restriction, and low birth weight [17–21]. Subclinical hyperthyroidism (defined as TSH values below the reference range with normal free T4 levels [22]), on the other hand, was not found to be associated with low birth weight, major malformations, or fetal, neonatal, or perinatal mortality in infants of 433 women with TSH levels ≤2.5th percentile and normal free T4 (≤1.75 ng/dL) relative to 23,124 women with normal TSH levels [23]. However, we are aware of no studies that investigated associations between high-normal T4 or subclinical hyperthyroidism and neurodevelopment.

The current study thus aims to examine whether maternal TH levels in the second half of pregnancy are associated with child neurodevelopment at 6, 12, 24, and 60 months of age. Prior studies used immunoassays to determine free T4 levels, but these measurements are influenced by T4-bound protein concentrations which increase during pregnancy [24]. In the present study, we used direct equilibrium dialysis to measure free T4 [25], a method that yields valid results in samples with normal or elevated T4-bound protein levels [26].

## 2. Methods

### 2.1. Participants

Pregnant women were recruited through the Center for the Health Assessment of Mothers and Children of Salinas (CHAMACOS), a birth cohort study of primarily Latino children born in the Salinas Valley, California. Women were eligible for inclusion in the study if they were ≥18 years old, had completed <20 weeks' gestation, spoke English or Spanish, were Medi-Cal eligible (state-sponsored health care for low-income families), were planning to deliver at the Monterey county hospital (Natividad Medical Center), and received prenatal care in this hospital or at one of five clinics of Clinica de Salud del Valle de Salinas. Screening and enrollment occurred between October 1999 and October 2000. We obtained informed consent from the 601 women who agreed to participate. Out of the 526 singleton live births (there were 20 miscarriages, 3 stillbirths, 2 neonatal deaths, 5 twin births, and 45 women were lost to follow-up), we excluded children with conditions that may impact scores on neurodevelopmental tests such as hydrocephaly (*n* = 1), autism (*n* = 1), and a history of seizures (*n* = 7). Children whose neurodevelopment was never assessed (*n* = 139) or whose mother's banked serum volume was insufficient for TH analysis (*n* = 91) were also excluded, leaving a final sample of 287 mother-child pairs. A total of 271 children were included at 6 months, 258 at 12 months, 240 at 24 months, and 207 at 60 months of age.

Women who were excluded or who dropped out at one or more time-point were more likely to be employed, to have been born in the US, to be depressed, and had lived longer in the US compared to those who were included in analyses. Excluded children were more likely to be firstborns and to have had lower birth weights compared to those who were included in the analyses. This study was approved by the University of California, Berkeley Committee for the Protection of Human Subjects.

### 2.2. Interviews

Women were interviewed in English or Spanish by bilingual, bicultural staff during pregnancy (at 13 and 27 weeks' gestation on average), at delivery, and when their children were 6, 12, 24, and 60 months of age. We obtained information about sociodemographic and lifestyle characteristics at each interview, including smoking, alcohol consumption, drug use, and diet during pregnancy; and on childcare, breastfeeding, number of children in the home, and housing density (number of people per room) after birth. The Peabody Picture Vocabulary Test (PPVT; at the 6 month visit) [27] and the Center for Epidemiologic Studies Depression Scale (CES-D; at the 12 month visits) [28] were also administered to mothers. In addition, the Infant-Toddler Home Observation for Measurement of the Environment (HOME) [29] was completed at 6 and 12 months; some subscales of the HOME were completed at 24 months. We also administered the Kotelchuck Adequacy of Prenatal Care Utilization Index [30], the Duke-UNC Functional Social Support Questionnaire [31], and the Diet Quality Index proposed by Bodnar and Siega-Riz [32] and modified by Harley and Eskenazi [33]. Mothers' (during pregnancy) and children's (up to age 24 months) medical records were abstracted by a registered nurse. We obtained data on delivery complications including vacuum extraction, placental abruption, amnionitis, and hemorrhage, or other bleeding; and on neonatal TSH levels (see below).

### 2.3. Neurodevelopmental Evaluations

Children were evaluated at the ages of 6, 12, 24, and 60 months. We selected for analyses those tests that assessed the same constructs examined in previous studies of maternal thyroid hormone and child neurodevelopment [2–7, 9–11]. Children were assessed at 6, 12, and 24 months of age on the Mental Development Index (MDI) and Psychomotor Development Index (PDI) of the Bayley Scales of Infant Development-Second Edition [34], and at 6 and 12 months on the auditory and expressive comprehension subscales of the Preschool Language Scale (PLS). At 60 months of age, the Performance Intellectual Quotient (IQ) was evaluated using the Wechsler Preschool and Primary Scale of Intelligence 3rd edition (WPPSI-III) [35]. We also administered the Vocabulary subtest of the WPPSI-III. Motor and language development, memory, attention, and school readiness were assessed using the McCarthy Scales of Children's Abilities (Digit Span Forward and Backward, Words and Sentences, Draw-a-Child, and Gross Motor Leg and Arm) [36], the Woodcock-Johnson Test of Cognitive Ability (Letter-Word and Applied Problems) and its Spanish-validated version (the Woodcock-Muñoz Test [37, 38]), the Pegboard subtest of the Wide Range Assessment of Visual-Motor Abilities (WRAVMA) for both dominant and non-dominant hands [39], the Conner's Kiddie Continuous Performance Test (KCPT) [40], and the PPVT [27]. The mother was queried about her child's behavior using the Child Behavior Checklist (CBCL). The Bayley Scales, the WPPSI performance IQ, the WRAVMA, the Woodcock-Johnson/Muñoz test, and the PPVT are age-standardized to a mean of 100 and a standard deviation of 15. Standardized scores on the McCarthy Scales are only available for full subscales but not for individual subtests. Raw scores were thus used for gross motor subtests. Scores on other McCarthy subtests were determined by subtracting children's chronological age from their developmental age (in months), as determined using methods published by Kaufman and Kaufman [41]. Positive scores show accelerated development while negative scores represent a delay. Finally, raw scores were used for the CBCL following recommendations from the test manual [42]. 

Neurodevelopmental evaluations were conducted in Spanish and/or English by psychometricians blind to mothers' TH levels in the study office or in a recreational vehicle (RV) modified for this purpose. Psychometricians were trained and supervised by a child neuropsychologist (CJ) and were videotaped and evaluated on a regular basis to ensure consistency across psychometricians and over time. All tests were reviewed by graduate students trained by the child neuropsychologist to ensure accurate scoring.

### 2.4. Thyroid Hormone Measurements

We measured TSH, free T4 and total T4 in serum collected by venipuncture from pregnant women at the time of the second interview (Mean ±  SD  =  26.9  ±  3.4 weeks' gestation). Samples were processed immediately at Natividad Medical Center and stored at −80°C at the UC Berkeley School of Public Health Biorepository until shipment to Quest Diagnostic's Nichols Institute (San Juan Capistrano, CA) where they were analyzed on a Bayer ADVIA Centaur system (Siemens Healthcare Diagnostics, Deerfield, IL). A pilot experiment revealed that every freeze-thaw cycle was associated with a 0.1 ng/dL increase in free T4 levels (*P* < 0.001); this variable accounted for 33% of the variance (unpublished results). Samples were thus thawed only once for aliquoting, shipped refrigerated, and analyzed within 48 hours. TSH was measured by ultrasensitive third generation immunochemiluminometric assay (ICMA; functional sensitivity (FS): 0.01 mIU/L, intra-assay coefficients of variation (CV) = 2.3–6.0%); total T4 was determined by solid-phase ICMA (FS: 0.1 *μ*g/dL, CV: 4.5–5.7%); free T4 was analyzed by direct equilibrium dialysis (ED) followed by radioimmunoassay (RIA; FS: 0.1 ng/dL, CV: 2.4–6.2%) [25]. Serum protein-bound T4 levels usually increase during pregnancy [15], which may bias results obtained by immunoassays not preceded by ED [24]. ED uses a semipermeable membrane to physically separate the bound hormone from the free portion, which is then measured using a highly sensitive RIA. This method measures free T4 accurately in samples with normal or elevated protein-bound T4 levels [26]. Previous studies used butanol-extractable iodine [2–4], which estimates T4 levels by measuring protein-bound iodine [43] or immunoassays [5–10]. Trimester-specific reference ranges for TH levels were provided by the analytical laboratory. Neonatal TSH was also measured in dried blood spots by the Genetics Disease Branch of the California Department of Health Services as part of the State's Newborn Screening Program. Hospital staff collected blood spots by heel stick on average 24.8 hours after birth (SD = 15.5); samples were analyzed by solid-phase, time-resolved sandwich fluoroimmunoassay (AutoDELPHIA; PerkinElmer, Wellesley, MA).

### 2.5. Statistical Analyses

Multiple linear regression models were used to evaluate associations between TH and neurodevelopmental outcomes. Models were first run with TH expressed continuously. We also ran models with TSH categorized as low (*n* = 43) versus normal based on trimester-specific reference ranges provided by the analytical laboratory. There were however not enough women with high TSH or with other TH measurements outside of the reference range to conduct such analyses. Therefore, to obtain sufficient sample size and to replicate methods used in prior studies [7–10], we dichotomized TH at the 10th and the 90th percentile based on distributions in our sample and at 0.8 ng/dL. Neurodevelopmental scores were expressed continuously. We used generalized additive models with a 3-degrees-of-freedom cubic spline function to evaluate the shape of the relationship between continuously expressed TH and scores on neurodevelopmental assessments and to test for linearity [44]. Since altered neurodevelopment was hypothesized to occur at both ends of the distribution of TH values (i.e., following an inverse U-shaped association), scores with *P* values for digression from linearity <0.10 were fit using a quadratic term while scores with a *P* value ≥0.10 were fit linearly in multiple regression models. Conclusions were similar when using quadratic or linear terms. We therefore only present results using linear terms. 

We removed outliers as identified by the Generalized Extreme Studentized Deviates Many-Outlier procedure at an *α* = 0.01 [45]. Covariates considered for inclusion in models were identified based on prior reports suggesting that they influenced neurodevelopment (see Appendix A for a complete list). They included (categorized as shown in Table 1 or as indicated below): maternal age at enrollment (continuously), race, education, income, parity (continuously), depression (yes versus no), maternal PPVT score (continuously), Diet Quality Index (continuously), Kotelchuck Adequacy of Prenatal Care Utilization Index (adequate plus, adequate, inadequate), Composite Social Support Index (continuously), employment status at the time of and prior to assessments (yes versus no), smoking (yes versus no), alcohol (yes versus no) and illegal drug (yes versus no) consumption during pregnancy, delivery type (natural versus cesarean section), pregnancy complications (any versus none), infant sex, premature birth (yes versus no), months of breastfeeding (continuously), HOME score at the time of and prior to assessments, and psychometrician administering assessments. 

To ensure that neurodevelopment was not affected by neonatal hypothyroidism, we also considered neonatal TSH levels as a covariate. In addition, we considered the potential confounding effect of some known neurotoxicants. Lead was measured in maternal and cord blood samples using graphite furnace atomic absorption spectrophotometry. As exposure to organophosphate insecticides has been associated with altered neurodevelopment in this cohort of farmworker families [46, 47], this variable was also considered. Organophosphate insecticide exposure was assessed by measuring dialkyl phosphate metabolites in maternal urine collected at approximately 13 and 26 weeks' gestation by high-resolution gas chromatography-tandem mass spectrometry (HRGC/MS-MS) with isotope dilution quantification [48]. Measurements at the two time points were averaged and log10-transformed. For each time point, covariates were included in final models if they were associated with any of the TH measurements at *P* < 0.10 based on analysis of variance (ANOVA) or Pearson's correlations. 

In order to control for potential selection bias due to exclusion from analyses and/or loss to followup, we ran all models with and without weights determined as the inverse probability of inclusion in our samples at each time-point [49]. Probability of inclusion was determined based on multiple logistic regression models using covariates listed in the Statistical Analyses section as potential predictors. Model selection was performed using a Deletion-Substitution-Addition (DSA) algorithm, which finds the combination of variables (including interactions and polynomials) that minimizes cross-validated risk [50]. Results were similar with and without this adjustment; we present results without the adjustment. Missing covariates were imputed. In addition, two free T4 and two TSH values below the limit of detection (LOD) (0.1 ng/dL and 0.01 mIU/L, resp.) were imputed as half the LOD. Statistical significance was defined as *P* < 0.05 on two-tailed tests. TSH values were log_2_-transformed for all statistical analyses. Analyses were performed using Intercooled STATA, version 10.0 (StataCorp, College Station, TX) and R, version 2.6.1 (R Foundation for Statistical Computing, Vienna, Austria).

## 3. Results

Mothers were mostly low-income, Mexican-born, Spanish-speaking Latinas with a low level of education, and many were recent immigrants to the United States (Table 1). A large proportion of women (73.7%) lived in farmworker families. During pregnancy, smoking was rare in this population (5.2%), and only 2.6% of women had ≥1 serving of alcohol per week. Mothers' mean PPVT score was 88.0 (SD = 21.2).

Mean free and total T4 levels were 0.8 ng/dL (SD = 0.2) and 10.5 *μ*g/dL (SD = 1.5), respectively; the geometric mean for TSH was 1.2 mIU/L (GSD = 1.7). Nine (2.7%) women had low free T4 (<0.5 ng/dL) and 13 (3.9%) had low total T4 (<8.0 *μ*g/dL) levels. None of the women were hypothyroidic based on the reference range for TSH provided by the analytical laboratory (TSH > 5.2 mIU/L), but 16 were hypothyroidic using the criteria proposed by the National Academy of Clinical Biochemistry (TSH > 2.5 mIU/L) [51, 52]. Five women had high free T4 (>1.6 ng/dL), none had elevated total T4 (>17.8 and >20.1 **μ**g/dL in the second and third trimesters, resp.), and 43 had low TSH (<0.5 and <0.8 mIU/L in the second and third trimesters, resp.). All children had normal TSH levels at birth (<25 mIU/L).

Scores on neurodevelopmental scales when children were aged 6, 12, 24, and 60 months are shown in Tables 2 and 3. Except for a low MDI score at 24 months (mean = 86.1; SD = 12.0), Bayley and PLS scores were close to the expected mean (i.e., 100) at all time points. At 60 months of age, children performed well on fine motor tests but scored relatively low on cognitive (verbal and nonverbal), language, and memory tests. 

Table 4 shows associations between maternal thyroid hormone levels and child scores on the Bayley and Preschool Language scales at 6, 12, and 24 months of age. Associations between maternal free T4 and scores on the Bayley scales were consistently negative but none were statistically significant either in unadjusted (results not shown) or adjusted models. Associations between Bayley scores and total T4 were also generally negative but not statistically significant. Increasing maternal TSH was related to better performance on the Bayley MDI and on the auditory comprehension subscale of the PLS at 12 months but maternal thyroid hormone was not related to these constructs at later points. Maternal free and total T4 levels were not significantly associated with scores on the PLS. 

Maternal free T4, total T4, and TSH were not associated with performance on any tests of neurodevelopment in 60-month-old children (Table 5) with one exception: every doubling in TSH levels was associated with a 0.65 point decrease (95% CI = −1.26, −0.04) on the Attention Deficit Hyperactivity Disorder (ADHD) subscale of the CBCL, although there was no significant association between maternal TH levels and CBCL Attention Problems and Pervasive Developmental Problems scales nor on child's performance on the KCPT (results not shown). Categorizing each measure of TH at the 10th or 90th percentiles yielded no significant association; subclinical hyperthyroidism was also not related with outcomes.

## 4. Discussion

We found little evidence that TH levels measured around the 27th week of gestation in mothers of euthyroid infants living in an iodine-sufficient area [53] were associated with child neurodevelopment. Although increasing maternal TSH levels were associated with better performance on the Bayley MDI at 12 months, these results did not persist at 24 months. Similarly, a reduction in ADHD symptoms, as reported by mothers in 60-month-old children, was not supported by other measures of hyperactivity and/or inattention at this age (i.e., maternal report on the Attention Problems scale of the CBCL or child performance on the KCPT). Better Auditory Comprehension also was noted at 12 months but not on other tests of language (WPPSI Vocabulary and PPVT) at 60 months.

Our results are in agreement with those reported by Pop and colleagues, the only other group that examined associations between maternal TH levels during the second half of gestation and child neurodevelopment [8–10]. In these studies, authors reported no associations between free T4 levels measured at 24 and 32 weeks' gestation and infant and toddler development, but did find relations with maternal thyroid hormone measured earlier in pregnancy. Other studies that measured TH during the first half of pregnancy have also reported associations with child neurodevelopment [2–10] with a notable exception in the study by Oken et al., which found no association between maternal TSH and total T4 at 10 weeks' gestation and child cognition at 6 months and 3 years of age in a large study of 500 mothers and children dyads [11]. TH of maternal origin may thus be of particular importance to brain development before the onset of fetal thyroid function, which occurs around midgestation [54]. Evidence for the potential role of maternal TH before the onset of fetal thyroid function includes the detection of T4 in coelomic fluid as early as 6 weeks' gestation [55], the fact that nuclear T3 receptors were identified in the brain of 10 week old fetuses [56], and that T3 binding to these receptors was detected between 9 and 13 weeks' gestation [57].

This study has some limitations. Women who were excluded from analyses were more likely to be depressed and to give birth to children of lower birth weight. This may have introduced bias since these variables are related to both thyroid hormone levels and neurodevelopment. However, our results were not substantially altered after applying inverse probability of inclusion weights, suggesting that this potential bias may not explain our null finding. In addition, in our study, as well as in those of Pop and colleagues [8–10], most women were euthyroid. Hence, our findings do not preclude the possibility that more extreme maternal thyroid hormone levels in the latter half of pregnancy may influence fetal neurodevelopment. 

This study has a number of strengths. We examined a wide range of domains of behavior and neurodevelopment at multiple ages and examined maternal thyroid hormone using direct equilibrium dialysis, which is currently considered the gold standard method to measure free T4. Prior studies exclusively used immunoassays, which, according to the National Academy of Clinical Biochemistry, may only be considered as free T4 “estimates” [52]. Another strength of the present study is that we were able to consider, and control for, a large number of potential confounders, including exposure to neurotoxicants such as lead, cigarette smoke, and organophosphate insecticides [58]. In addition, our population is demographically homogenous, further reducing the potential for confounding. Finally, Zoeller and Rovet proposed that maternal hypothyroxinemia and hypothyroidism at the beginning of the third trimester (when we determined thyroid function in CHAMACOS women) primarily affects gross and fine motor skills, memory, and visuospatial skills [59]. In this study, we evaluated these constructs using well-validated and widely used instruments and yet found no clear evidence of a relationship between maternal thyroid hormone levels and child neurodevelopment. 

In summary, this is the first study of maternal thyroid hormone and child neurodevelopment to use direct equilibrium dialysis to measure free T4. Although prior studies did report associations between maternal clinical hypothyroidism and mild hypothyroxinemia during the first half of pregnancy and cognitive impairments in children, we find no convincing evidence that TH measured during later gestation is associated with neurodevelopment in euthyroid children living in an iodine-sufficient area.

## Figures and Tables

**Table 1:** Demographic characteristics of study participants (*n* = 287).

	No. (%)
*Mothers*	
Age (years)	
18–24	130 (45.3)
25–29	95 (33.1)
30–34	42 (14.6)
35–45	20 (7.0)

Race/Ethnicity	
White	5 (1.7)
Latino	278 (96.9)
Other	4 (1.4)

Education	
≤6th grade	121 (42.2)
7–12th grade	105 (36.6)
≥High School	61 (21.3)

Income (% poverty)	
<100	171 (59.6)
100–200	105 (36.6)
>200	11 (3.8)

Country of birth	
United States	32 (11.1)
Mexico	251 (87.5)
Other	4 (1.4)

Time in the USA (years)	
≤5	156 (54.4)
6–10	69 (24.0)
≥11	62 (21.6)

Parity	
0	91 (31.7)
≥1	196 (68.3)

Smoking during pregnancy	
No	271 (94.4)
Yes	16 (5.6)

Alcohol during pregnancy (≥one serving)	
No	282 (98.3)
Yes	5 (1.7)

*Children*	
Sex	
Boy	140 (48.8)
Girl	147 (51.2)

Birthweight (g)	
<2500 g	10 (3.5)
2500–3500 g	149 (51.9)
>3500 g	128 (44.6)

Gestational duration (weeks)	
<37	21 (7.3)
37–42	266 (92.7)
>42	0 (0.0)

**Table 2:** Mean scores on neurodevelopmental scales at 6, 12, and 24 months of age.

	6 Months		12 Months		24 Months	
	*N*	Mean (SD)	*N*	Mean (SD)	*N*	Mean (SD)
Bayley						
Mental development index	271	95.0 (7.9)	258	101.3 (9.1)	240	86.4 (11.7)
Psychomotor development index	271	96.2 (11.1)	257	107.0 (12.8)	240	98.2 (10.4)

Preschool language scale						
Auditory comprehension	270	104.6 (12.9)	257	99.1 (12.9)		
Expressive comprehension	270	91.1 (13.5)	257	94.5 (13.8)		
Total score	270	97.7 (11.3)	257	96.6 (13.5)		

**Table 3:** Mean scores on neurodevelopmental scales at 5 years of age.

	*N*	Mean (SD)
*Intelligence*		
WPPSI^1^		
Performance IQ	207	94.7 (14.7)

*Motor*		
WRAVMA^2^		
Pegboard-Dominant	205	110.7 (17.4)
Pegboard-Nondominant	204	110.3 (17.2)
McCarthy		
Draw-a-Child	206	3.9 (16.1)
Gross Motor-Leg	194	11.0 (2.2)
Gross Motor-Arm	202	4.1 (2.4)

*Language Development*		
WPPSI^1^ Vocabulary	207	8.8 (2.6)
PPVT^3^	205	94.8 (17.5)

*Memory*		
McCarthy		
Words and Sentences	205	−4.5 (16.6)
Digit Span Forward	204	−15.2 (13.0)
Digit Span Backward	199	−15.3 (10.6)

*School Readiness*		
Woodcock-Johnson/Muñoz		
Letter-Word	199	92.4 (12.1)
Applied Problems	206	87.0 (15.8)

*Attention*		
CBCL^4^		
ADHD^5^	200	4.7 (2.8)
KCPT^6^		
ADHD Confidence Index^5^	188	45.7 (17.5)

^1^Weschler Preschool and Primary Scale of Intelligence.

^2^Wide Range Assessment of Visual Motor Ability.

^3^Peabody Picture Vocabulary Test.

^4^Child Behavior Checklist.

^5^Attention Deficit Hyperactivity Disorder.

^6^Kiddie Continuous Performance Test.

Note: We report differences between chronological and developmental ages for the McCarthy Draw-a-Child, Words and Sentences, and Digit Span Forward and Backward subtests (in months). Raw scores are reported for the gross motor tasks of the McCarthy scales (no developmental ages are available for these subtests) and for the CBCL as recommended by the test manual [42]. Standardized scores are used for other tests.

**Table 4:** Associations between maternal thyroid hormone levels during pregnancy (27 weeks' gestation) and child neurodevelopment at 6, 12 and 24 months of age.

			Bayley scales		Preschool language scale		
			Mental development index	Psychomotor development index	Auditory comprehension	Expressive comprehension	Total score
6 Months^1^	Free T4	**β**	−3.14	−3.27	−2.03	1.65	−0.10
		(95% CI)	(−7.65, 1.36)	(−9.36, 2.82)	(−9.80, 5.75)	(−5.48, 8.79)	(−6.45, 6.25)
	Total T4	**β**	0.03	−0.06	−0.24	0.58	0.19
		(95% CI)	(−0.57, 0.62)	(−0.86, 0.74)	(−1.28, 0.80)	(−0.37, 1.53)	(−0.66, 1.05)
	TSH	**β**	0.85	1.46	1.51	0.24	1.06
		(95% CI)	(−0.49, 2.18)	(−0.33, 3.26)	(−0.81, 3.84)	(−1.91, 2.38)	(−0.87, 2.98)

12 Months^2^	Free T4	**β**	−0.48	−6.40	4.39	1.26	3.45
		(95% CI)	(−6.22, 5.26)	(−13.89, 1.08)	(−3.56, 12.34)	(−7.20, 9.72)	(−4.76, 11.67)
	Total T4	**β**	−0.12	−0.45	0.33	−0.15	0.12
		(95% CI)	(−0.92, 0.68)	(−1.53, 0.63)	(−0.79, 1.46)	(−1.34, 1.04)	(−1.05, 1.28)
	TSH	**β**	1.71	0.10	2.92	0.46	1.91
		(95% CI)	(0.05, 3.37)*	(−2.16, 2.35)	(0.59, 5.25)*	(−2.04, 2.95)	(−0.51, 4.33)

24 Months^3^	Free T4	**β**	−4.29	−2.67			
		(95% CI)	(−11.60, 3.02)	(−8.95, 3.62)			
	Total T4	**β**	−0.17	0.43			
		(95% CI)	(−1.20, 0.85)	(−0.43, 1.30)			
	TSH	**β**	0.33	−1.31			
		(95% CI)	(−1.97, 2.63)	(−3.25, 0.63)			

*P < 0.05.

^1^Models adjusted for maternal age, employment status at enrollment and at the 6-months visit, country of birth, time lived in the US, Diet Quality Index, blood lead levels and delivery complications; child hospitalization before 6 months, season of assessment and psychometrician.

^2^Models adjusted for maternal age, employment status at enrollment and at the 6 months visit, country of birth, time lived in the US, Diet Quality Index, Kotelchuck Adequacy of Prenatal Care Utilization Index, blood lead levels, delivery complications and PPVT score; child age, preterm birth, hospitalization at 6 months and 1 year, family structure at 1 year; season, and language spoken at the time of assessment.

^3^Models adjusted for maternal age, income, employment status at enrollment, 6 months and 1 year, country of birth, Diet Quality Index, Kotelchuck Adequacy of Prenatal Care Utilization Index, blood lead levels, delivery complications, PPVT score; child hospitalization at 1 year; number of children in the home at 6 months, home density at 2 years, family structure at 1 year; season, psychometrician and language of assessment.

^4^Since TSH was expressed on a log_2_ basis, **β** are equal to the change in neurodevelopmental outcomes for a doubling in TSH levels.

**Table 5:** Associations between maternal thyroid hormone levels during pregnancy (27 weeks' gestation) and child neurodevelopment at 5 years of age.^1^

	Free T4		Total T4		TSH^2^	
	**β**	(95% CI)	**β**	(95% CI)	**β**	(95% CI)
*Performance IQ*						
WPPSI^3^	−4.12	(−13.73, 5.49)	0.03	(−1.35, 1.41)	−2.26	(−5.27, 0.74)

*Motor Development*						
WRAVMA^4^						
Pegboard-Dominant	−3.76	(−15.45, 7.93)	−0.97	(−2.64, 0.70)	0.51	(−3.12, 4.15)
Pegboard-Nondominant	−4.21	(−16.03, 7.61)	−1.55	(−3.23, 0.13)	0.02	(−3.65, 3.68)
McCarthy						
Draw-a-Child	−5.98	(−16.74, 4.77)	0.10	(−1.44, 1.63)	0.06	(−3.27, 3.39)
Gross Motor-Leg	−0.14	(−1.60, 1.32)	0.00	(−0.22, 0.22)	0.16	(−0.31, 0.63)
Gross Motor-Arm	0.08	(−1.51, 1.66)	−0.04	(−0.27, 0.19)	0.04	(−0.45, 0.53)

*Language Development*						
WPPSI^3^ Vocabulary	−0.21	(−1.98, 1.57)	−0.22	(−0.47, 0.03)	−0.37	(−0.92, 0.19)
PPVT^5^	−2.71	(−14.18, 8.77)	−0.89	(−2.54, 0.76)	−1.05	(−4.66, 2.57)

*Memory*						
McCarthy						
Words and Sentences	0.10	(−11.32, 11.52)	−0.55	(−2.17, 1.07)	−1.44	(−4.97, 2.09)
Digit Span Forward	4.06	(−4.72, 12.83)	0.77	(−0.46, 2.00)	−1.83	(−4.51, 0.86)
Digit Span Backward	4.43	(−2.55, 11.42)	−0.10	(−1.13, 0.94)	−0.38	(−2.63, 1.87)

*School Readiness*						
Woodcock-Johnson/Muñoz						
Letter-Word	−1.86	(−9.33, 5.60)	0.21	(−0.88, 1.31)	1.43	(−0.94, 3.80)
Applied Problems	−3.82	(−14.06, 6.42)	−0.51	(−2.01, 1.00)	−1.48	(−4.74, 1.78)

*Attention*						
CBCL^6^						
ADHD^7^	−0.10	(−2.03, 1.82)	0.00	(−0.28, 0.27)	−0.65	(−1.26, −0.04)*
KCPT^8^						
ADHD^7^ Confidence Index	7.52	(−4.86, 19.91)	0.09	(−1.70, 1.87)	−0.75	(−4.61, 3.12)

*P < 0.05.

^1^Models adjusted for maternal age, income, employment status at 6 months, country of birth, Diet Quality Index, delivery complications, PPVT score; child 5-minute APGAR, hospitalization at 1 year; number of children in home at 1 and 2 years, home density at 2 years, family structure at 1 year; season of assessment.

^2^Since TSH was expressed on a log_2_ basis, **β** are equal to the change in neurodevelopmental outcomes for a doubling in TSH levels.

^3^Weschler Preschool and Primary Scale of Intelligence.

^4^Wide Range Assessment of Visual Motor Ability.

^5^Peabody Picture Vocabulary Test.

^6^Child Behavior Checklist.

^7^Attention Deficit Hyperactivity Disorder.

^8^Kiddie Continuous Performance Test.

## Appendices

### A. Maternal Covariates

**Table:**

	
*Baseline visit*	
Age at enrollment (years), No. (%)	
18–24	130 (45.3)
25–29	95 (33.1)
30–34	42 (14.6)
34–45	20 (7.0)
Age at enrollment (years), Mean (SD)	25.8 (5.0)
Race, No. (%)	
White	5 (1.7)
Latino	278 (96.9)
Other	4 (1.4)
Education, No. (%)	
≤6th grade	121 (42.2)
7–12th grade	105 (36.6)
≥High School	61 (21.3)
Income (% poverty), No. (%)	
<100	171 (59.6)
100–200	105 (36.6)
>200	11 (3.8)
Average income per person per month ($), Mean (SD)	413.8 (255.7)
Employment status, No. (%)	
No	209 (72.8)
Yes	78 (27.2)
Country of birth, No. (%)	
United States	32 (11.1)
Mexico	251 (87.5)
Other	4 (1.4)
Time in the USA (years), No. (%)	
≤5	156 (54.4)
6 to 10	69 (24.0)
≥11	62 (21.6)
Parity, Mean (SD)	1.3 (1.2)
Smoking during pregnancy, No. (%)	
No	271 (94.4)
Yes	16 (5.6)
Smokers in household during pregnancy, No. (%)	
No	258 (89.9)
Yes	29 (10.1)
Any second-hand smoke exposure during pregnancy, No. (%)	
No	179 (62.4)
Yes	108 (37.6)
More than one alcoholic drink per week during pregnancy, No. (%)	
No	282 (98.3)
Yes	5 (1.7)
Any drug consumption during pregnancy, No. (%)	
No	282 (98.3)
Yes	5 (1.7)
Kotelchuck Adequacy of Prenatal Care Utilization Index, No. (%)	
Inadequate	62 (21.6)
Adequate	93 (32.4)
Adequate Plus	132 (46.0)
Diet Quality Index during pregnancy, Mean (SD)	45.3 (9.7)
Composite Social Support Index, Mean (SD)	3.7 (0.9)
Urinary DAP^1^metabolites during pregnancy (nmol/L), Mean (SD)	2.1 (0.4)
Lead levels during pregnancy (ug/dL), Mean (SD)	1.5 (2.1)
*6-Month Visit*	
Income (% poverty), No. (%)	
<100	199 (69.3)
100–200	86 (30.0)
>200	2 (0.7)
Employment status, No. (%)	
No	199 (69.3)
Yes	88 (30.7)
PPVT^2^ score, Mean (SD)	88.2 (21.1)
WAIS^3^ score, Mean (SD)	6.3 (2.6)
*12-Month Visit*	
Income (% poverty), No. (%)	
<100	179 (62.4)
100–200	99 (34.5)
>200	9 (3.1)
Employment status, No. (%)	
No	198 (69.0)
Yes	89 (31.0)
Composite Social Support Index, Mean (SD)	3.8 (1.0)
Depression, No. (%)	
No	140 (48.8)
Yes	147 (51.2)
*24-Month Visit*	
Income (% poverty), No. (%)	
<100	167 (58.2)
100–200	107 (37.3)
>200	13 (4.5)
Employment status, No. (%)	
No	174 (60.6)
Yes	113 (39.4)
Composite Social Support Index, Mean (SD)	3.9 (1.0)
*Three-Year Visit*	
Income (% poverty), No. (%)	
<100	178 (62.0)
100–200	103 (35.9)
>200	6 (2.1)
Employment status, No. (%)	
No	175 (61.0)
Yes	112 (39.0)
Depression, No. (%)	
No	161 (56.1)
Yes	126 (43.9)
*60-Month visit*	
Income (% poverty), No. (%)	
<100	182 (63.4)
100–200	93 (32.4)
>200	12 (4.2)
Employment status, No. (%)	
No	158 (55.1)
Yes	129 (44.9)
Composite Social Support Index, Mean (SD)	3.9 (1.0)

^1^Dialkyl phosphates (DAPs) measured in maternal urine (organophosphate pesticide metabolites).

^2^Peabody Picture Vocabulary Test (PPVT).

^3^Wechsler Adult Intelligence Scale (WAIS).

### B. Child Covariates

**Table:**

	
*Baseline visit*	
Sex, No. (%)	
Boy	140 (48.8)
Girl	147 (51.2)
Birthweight (g), Mean (%)	
<2500	10 (3.5)
2500–3500	149 (51.9)
>3500	128 (44.6)
Gestational duration (weeks), No. (%)	
≥37	266 (92.7)
<37	21 (7.3)
Cesarean section, No. (%)	
No	220 (76.7)
Yes	67 (23.3)
Pregnancy complications, No. (%)	
No	284 (99.0)
Yes	3 (1.0)
5-minute APGAR score, Mean (SD)	8.9 (0.4)
Months child breastfed, Mean (SD)	8.6 (8.2)
Neonatal TSH (mIU/L), Mean (SD)	6.5 (3.5)
*6-Month Visit*	
Number of children in household, Mean (SD)	2.1 (0.4)
Housing density (people per room), No. (%)	
≤0.5	4 (1.4)
0.51–1.00	52 (18.1)
1.01–1.50	93 (32.4)
≥1.51	138 (48.1)
Lived with father, No. (%)	
All the time	242 (84.3)
Most of the time	11 (3.8)
Some of the time	14 (4.9)
Not at all	20 (7.0)
HOME^1^ score, Mean (SD)	32.0 (4.1)
Hospitalized overnight, No. (%)	
No	253 (88.2)
Yes	34 (11.8)
Age at assessment (months), Mean (SD)	6.6 (1.1)
Medication/herbal intake within 24 hours of assessment, No. (%)	
No	279 (97.2)
Yes	8 (2.8)
Location assessment performed, No. (%)	
Office	187 (65.2)
Other	100 (34.8)
Season assessment performed, No. (%)	
January–March	68 (23.7)
April–June	71 (24.7)
July–September	77 (26.8)
October–December	71 (24.7)
Psychometrician at assessment, No. (%)	
01	117 (40.8)
07	32 (11.1)
13	42 (14.6)
16	4 (1.4)
23	92 (32.1)
*12-Month Visit*	
Number of children in household, Mean (SD)	2.0 (1.7)
Housing density (people per room), No. (%)	
≤0.5	1 (0.3)
0.51–1.00	55 (19.2)
1.01–1.50	105 (36.6)
≥1.51	126 (43.9)
Lived with father, No. (%)	
All the time	239 (83.3)
Most of the time	11 (3.8)
Some of the time	13 (4.5)
Not at all	24 (8.4)
HOME^1^ score, Mean (SD)	35.9 (3.1)
Hospitalized overnight, No. (%)	
No	276 (96.2)
Yes	11 (3.8)
Age at assessment (months), Mean (SD)	12.7 (1.3)
Medication/herbal intake within 24 hours of assessment, No. (%)	
No	255 (88.9)
Yes	32 (11.1)
Location assessment performed, No. (%)	
Office	190 (66.2)
Other	97 (33.8)
Season assessment performed, No. (%)	
January–March	73 (25.4)
April–June	67 (23.3)
July–September	74 (25.8)
October–December	73 (25.4)
Psychometrician at assessment, No. (%)	
01	109 (38.0)
07	83 (28.9)
23	95 (33.1)
*24-Month visit*	
Number of children in household, Mean (SD)	1.8 (1.5)
Housing density (people per room), No. (%)	
≤0.5	2 (0.7)
0.51–1.00	51 (17.8)
1.01–1.50	94 (32.8)
≥1.51	140 (48.8)
Lived with father, No. (%)	
All the time	236 (82.2)
Most of the time	23 (8.0)
Some of the time	5 (1.7)
Not at all	23 (8.0)
HOME^1^ score, Mean (SD)	26.1 (2.5)
Hospitalized overnight, No. (%)	
No	275 (95.8)
Yes	12 (4.2)
Age at assessment (months), Mean (SD)	24.7 (1.2)
Medication/herbal intake within 24 hours of assessment, No. (%)	
No	205 (71.4)
Yes	82 (28.6)
Location assessment performed, No. (%)	
Office	194 (67.6)
Other	93 (32.4)
Season assessment performed, No. (%)	
January–March	73 (25.4)
April–June	85 (29.6)
July–September	71 (24.7)
October–December	58 (20.2)
Psychometrician at assessment, No. (%)	
01	122 (42.5)
07	31 (10.8)
23	134 (46.7)
*60-Month visit*	
Number of children in household, Mean (SD)	1.9 (1.3)
Housing density (people per room), No. (%)	
≤0.5	1 (0.3)
0.51–1.00	87 (30.3)
1.01–1.50	126 (43.9)
≥1.51	73 (25.4)
Lived with father, No. (%)	
All the time	221 (77.0)
Most of the time	16 (5.6)
Some of the time	11 (3.8)
Not at all	39 (13.6)
Hospitalized overnight, No. (%)	
No	279 (97.2)
Yes	8 (2.8)
Attended preschool, No. (%)	
No	124 (43.2)
Yes	163 (56.8)
Attended kindergarten, No. (%)	
No	68 (23.7)
Yes	219 (76.3)
Age at assessment (months), Mean (SD)	60.7 (2.2)
Medication/herbal intake within 24 hours of assessment, No. (%)	
No	240 (83.6)
Yes	47 (16.4)
Location assessment performed, No. (%)	
Office	245 (85.4)
Other	42 (14.6)
Season assessment performed, No. (%)	
January–March	76 (26.5)
April–June	72 (25.1)
July–September	86 (30.0)
October–December	53 (18.5)
Psychometrician at assessment, No. (%)	
01	117 (40.8)
21	23 (8.0)
23	32 (11.1)
43	115 (40.1)

^1^Home Observation for Measurement of the Environment (H.O.M.E).
